# Supplementary material for: Role and mechanism of NCAPD3 in promoting malignant behaviors in gastric cancer
Source: Front Pharmacol. 2024 Apr 22;15:1341039. doi: 10.3389/fphar.2024.1341039 (PMC11070777; doi:10.3389/fphar.2024.1341039)
Supplement: Supplementary file 11 [file DataSheet2.ZIP › GSEA/GO_CC/my_analysis.Gsea.1599461408869/GO_EXTERNAL_SIDE_OF_PLASMA_MEMBRANE.html]

Details for gene set GO\_EXTERNAL\_SIDE\_OF\_PLASMA\_MEMBRANE[GSEA]

|  || Dataset | filtered\_dataset.sample\_info.cls#WT\_versus\_NCAPD3\_MUT |
| Phenotype | sample\_info.cls#WT\_versus\_NCAPD3\_MUT |
| Upregulated in class | NCAPD3\_MUT |
| GeneSet | GO\_EXTERNAL\_SIDE\_OF\_PLASMA\_MEMBRANE |
| Enrichment Score (ES) | -0.36455914 |
| Normalized Enrichment Score (NES) | -1.8536558 |
| Nominal p-value | 0.0024937657 |
| FDR q-value | 0.062764645 |
| FWER p-Value | 0.282 |
Table: GSEA Results Summary

  

Fig 1: Enrichment plot: GO\_EXTERNAL\_SIDE\_OF\_PLASMA\_MEMBRANE      
 Profile of the Running ES Score & Positions of GeneSet Members on the Rank Ordered List

  

| SYMBOL | TITLE | RANK IN GENE LIST | RANK METRIC SCORE | RUNNING ES | CORE ENRICHMENT || 1 | 2243 | FGA | 18 | 1.039 | 0.0666 | No |
| 2 | 2244 | FGB | 321 | 0.582 | -0.1067 | No |
| 3 | 1755 | DMBT1 | 480 | 0.487 | -0.1834 | No |
| 4 | 7099 | TLR4 | 541 | 0.451 | -0.1921 | No |
| 5 | 57493 | HEG1 | 683 | 0.386 | -0.2643 | No |
| 6 | 1605 | DAG1 | 823 | 0.304 | -0.3413 | Yes |
| 7 | 7037 | TFRC | 824 | 0.303 | -0.3180 | Yes |
| 8 | 3673 | ITGA2 | 858 | -0.264 | -0.3216 | Yes |
| 9 | 8140 | SLC7A5 | 890 | -0.305 | -0.3206 | Yes |
| 10 | 301 | ANXA1 | 914 | -0.342 | -0.3109 | Yes |
| 11 | 3949 | LDLR | 929 | -0.353 | -0.2940 | Yes |
| 12 | 5621 | PRNP | 970 | -0.387 | -0.2932 | Yes |
| 13 | 9180 | OSMR | 981 | -0.394 | -0.2702 | Yes |
| 14 | 9308 | CD83 | 1025 | -0.423 | -0.2688 | Yes |
| 15 | 966 | CD59 | 1040 | -0.436 | -0.2455 | Yes |
| 16 | 1889 | ECE1 | 1091 | -0.468 | -0.2457 | Yes |
| 17 | 3134 | HLA-F | 1139 | -0.506 | -0.2408 | Yes |
| 18 | 2152 | F3 | 1188 | -0.559 | -0.2326 | Yes |
| 19 | 7057 | THBS1 | 1242 | -0.620 | -0.2234 | Yes |
| 20 | 154064 | RAET1L | 1276 | -0.664 | -0.1963 | Yes |
| 21 | 80328 | ULBP2 | 1277 | -0.664 | -0.1453 | Yes |
| 22 | 353091 | RAET1G | 1278 | -0.664 | -0.0944 | Yes |
| 23 | 4240 | MFGE8 | 1280 | -0.668 | -0.0439 | Yes |
| 24 | 79465 | ULBP3 | 1366 | -0.847 | -0.0404 | Yes |
| 25 | 5156 | PDGFRA | 1383 | -0.932 | 0.0195 | Yes |
Table: GSEA details [plain text format]

  

Fig 2: GO\_EXTERNAL\_SIDE\_OF\_PLASMA\_MEMBRANE      
 Blue-Pink O' Gram in the Space of the Analyzed GeneSet

  

Fig 3: GO\_EXTERNAL\_SIDE\_OF\_PLASMA\_MEMBRANE: Random ES distribution      
 Gene set null distribution of ES for **GO\_EXTERNAL\_SIDE\_OF\_PLASMA\_MEMBRANE**
